# Antegrade horizontal stenting for endovascular coiling of wide-neck aneurysms in the basilar tip: Two case reports

**DOI:** 10.1097/MD.0000000000041451

**Published:** 2025-01-31

**Authors:** Byung Hyun Baek, Woong Yoon, You Sub Kim, Tae Sun Kim, Seul Kee Kim, Yun Young Lee, Byung Chan Lee, Chan Park

**Affiliations:** a Department of Radiology, Chonnam National University Hospital and Chonnam National University Medical School, Dong-gu, Gwangju, Republic of Korea; b Department of Neurosurgery, Chonnam National University Hospital and Chonnam National University Medical School, Dong-gu, Gwangju, Republic of Korea; c Department of Radiology, Chonnam National University Hwasun Hospital and Chonnam National University Medical School, Hwasun-eup, Hwasun-gun, Jeollanam-do, Republic of Korea.

**Keywords:** antegrade, basilar tip, horizontal stenting, jailed microcatheter, wide-neck aneurysm

## Abstract

**Rationale:**

Endovascular coil embolization for wide-neck intracranial aneurysms is technically challenging and requires supporting devices. Intracranial stents are widely used as a buttress for compact coil filling and preventing coil protrusion into the parent artery. Horizontal stenting has been developed to maximize coil insertion and reduce the risk of thromboembolism. An anterograde route for horizontal stenting could be attempted for basilar tip aneurysms if the posterior communicating artery is not accessible. Here, we report 2 cases of antegrade horizontal stenting (AHS) for wide-neck aneurysm (WNA) in the basilar tip. This approach aims to maximize coil insertion and achieve complete aneurysm occlusion.

**Patient concerns:**

Both patients were admitted for the treatment of cerebral aneurysms, which were incidentally detected during routine health checkups.

**Diagnoses:**

Both patients were diagnosed with WNAs at the basilar tip and hypoplastic communicating arteries.

**Interventions:**

The patients underwent stent-assisted coil embolization for WNA in the basilar tip using the catheter jailing technique. After positioning a pre-jailed microcatheter within a basilar aneurysm, AHS was performed using the Neuroform Atlas stent, with the proximal part of the stent deployed across the neck of the basilar tip aneurysm. In both cases, the preselected microcatheter tip shifted either to the anterior or posterior side of the aneurysm immediately after AHS using the pushing technique. The coils inserted into the aneurysm did not reach the opposite side of the microcatheter placement, necessitating additional catheterization.

**Outcomes:**

Complete occlusion was achieved by inserting additional coils through the repositioned microcatheter.

**Lessons:**

Horizontal stenting via an antegrade route may be an effective strategy for achieving compact coil embolization in selected patients with WNAs at the basilar tip. The Neuroform Atlas stent appears to be a promising tool for AHS owing to its hybrid-cell design. However, the shifting phenomenon of a jailed microcatheter after AHS warrants attention, as it may present potential challenges that require further catheterization.

## 1. Introduction

Endovascular coil embolization for wide-neck intracranial aneurysms is technically challenging and requires supporting techniques. Stent-assisted coiling, balloon assisted coiling, coiling with multiple catheters, and parent artery complex coil protection are used adjunctively to ensure adequate coil packing.^[1,2]^ Among these techniques, intracranial stents are widely used as a buttress for compact coil filling and preventing coil protrusion into the parent artery. Stent-assisted coiling is an effective method and is associated with a decrease in recurrences.^[3]^

Furthermore, horizontal stenting, Y-stenting, and waffle cone stent-assisted coiling have been proposed to cover complicated wide-neck aneurysms to prevent coil protrusion.^[1,2,4]^ Horizontal stenting has been developed to maximize coil insertion and reduce the risk of thromboembolism.^[4]^ Retrograde or transcirculation routes for horizontal stenting can only be applied in patients with accessible communicating arteries. The jailed double-microcatheter technique with horizontal stenting was introduced for the treatment of wide-neck aneurysms, resulting in immediate complete obliteration.^[5]^ An anterograde route for horizontal stenting could be attempted for basilar tip aneurysms if the posterior communicating artery is not accessible.^[6]^ In this report, we describe antegrade horizontal stenting using the Neuroform Atlas stent (Stryker Neurovascular, Fremont, CA, USA) for coil embolization in 2 cases of wide neck aneurysms in the basilar tip. Furthermore, we focus on the suitability of the Neuroform Atlas stent for antegrade horizontal stenting and the shifting phenomenon of jailed microcatheter following stenting.

## 2. Case presentation

### 2.1. Case 1

A 76-year-old woman was admitted to our hospital for the treatment of incidentally detected aneurysms in the basilar tip and A3 segment of the anterior cerebellar artery (ACA). Digital subtraction angiography (DSA) revealed a basilar tip aneurysm with a width of 5.4 mm and a neck of 5.2 mm. Our plan was to perform stent-assisted coil embolization for the treatment of the basilar tip aneurysm. Both posterior communicating arteries were hypoplastic. Additionally, a right ACA aneurysm with a length of 10 mm and a neck of 3 mm was deemed suitable for simple coil embolization due to its narrow neck. The patient was prescribed a daily dose of 75 mg of clopidogrel and 100 mg of aspirin for 3 days.

**Figure 1.**
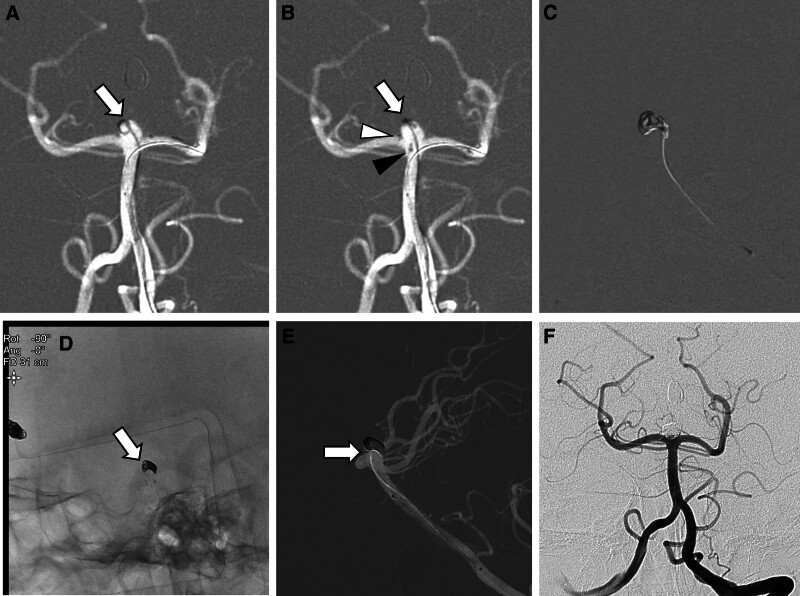
Shifting phenomenon of the jailed microcatheter posteriorly after horizontal stenting. (A) Partially deployed coils (arrow) using a jailed microcatheter and a stent-loaded microcatheter in the left P1. (B) Note the proximal stent markers (arrowhead) fanned out in the right P1, the tip of the microcatheter (black arrowhead) after releasing the stent, and shifted jailed microcatheter containing a predeployed coil (arrow) immediately after horizontal stenting. (C) Lateral view showing the microcatheter tip (arrow) shifted towards the rear of the deployed stent and coils packed in the posterior part of the aneurysm. (D) Insufficient coil filling in the anterior part of the aneurysm (arrow). *Note*: The prior coiled aneurysm at right anterior cerebral artery. (E) Additional coils inserted through the repositioned microcatheter with an anteriorly located tip (arrow). (F) Final angiography showing complete occlusion.

Under general anesthesia, a 6-Fr Chaperon guiding catheter was placed in the right internal carotid artery, and an Excelsior SL-10 microcatheter (Stryker Neurovascular, Fremont, CA) was guided into the right ACA aneurysm using a Traxcess-14 microguidewire (MicroVention, Tustin, CA). Simple coil embolization was then performed. After the successful treatment of the ACA aneurysm, the 6-Fr Chaperon guiding catheter was repositioned in the left vertebral artery. An SL-10 microcatheter was inserted into the aneurysm, and a partial frame was created using the first coil. For the jailed technique, a Headway 17 microcatheter (MicroVention, Aliso Viejo, CA) was navigated to the P1 segment of the left posterior cerebellar artery (PCA) to deliver the 3 × 15 mm Neuroform Atlas stent (Fig. 1A). After deploying the horizontal stent from left P1 to right P1, across the aneurysmal neck, the jailed microcatheter was shifted, and a predeployed coil loop was tilted (Fig. 1B). Coiling was then performed through the microcatheter positioned posterior to the deployed stent (Fig. 1C). The framing and filling coils were primarily inserted in the posterior part of the aneurysm. A lateral view revealed insufficient coil filling in the anterior part of the aneurysm (Fig. 1D). The microcatheter was easily repositioned to the anterior part of the aneurysm using the jailed approach, and complete exclusion of the aneurysm was successfully achieved through coiling with the repositioned microcatheter, ensuring that no coil protrusion into the basilar artery. The lateral view demonstrated the buttress of the stent and fully packed coils gripping the stent after coiling (Fig. 1E). The aneurysm was completely occluded (Fig. 1F). A total of 2000 U of heparin was administered during the procedure, and the patient was also given dual antiplatelet medications. Diffusion-weighted MR images taken 1 day after the procedure showed no ischemic changes in the midbrain and thalamus, except for a small, clinically silent ischemic lesion in the cerebellar vermis. The patient remained asymptomatic and was discharged 3 days after the procedure.

### 2.2. Case 2

**Figure 2.**
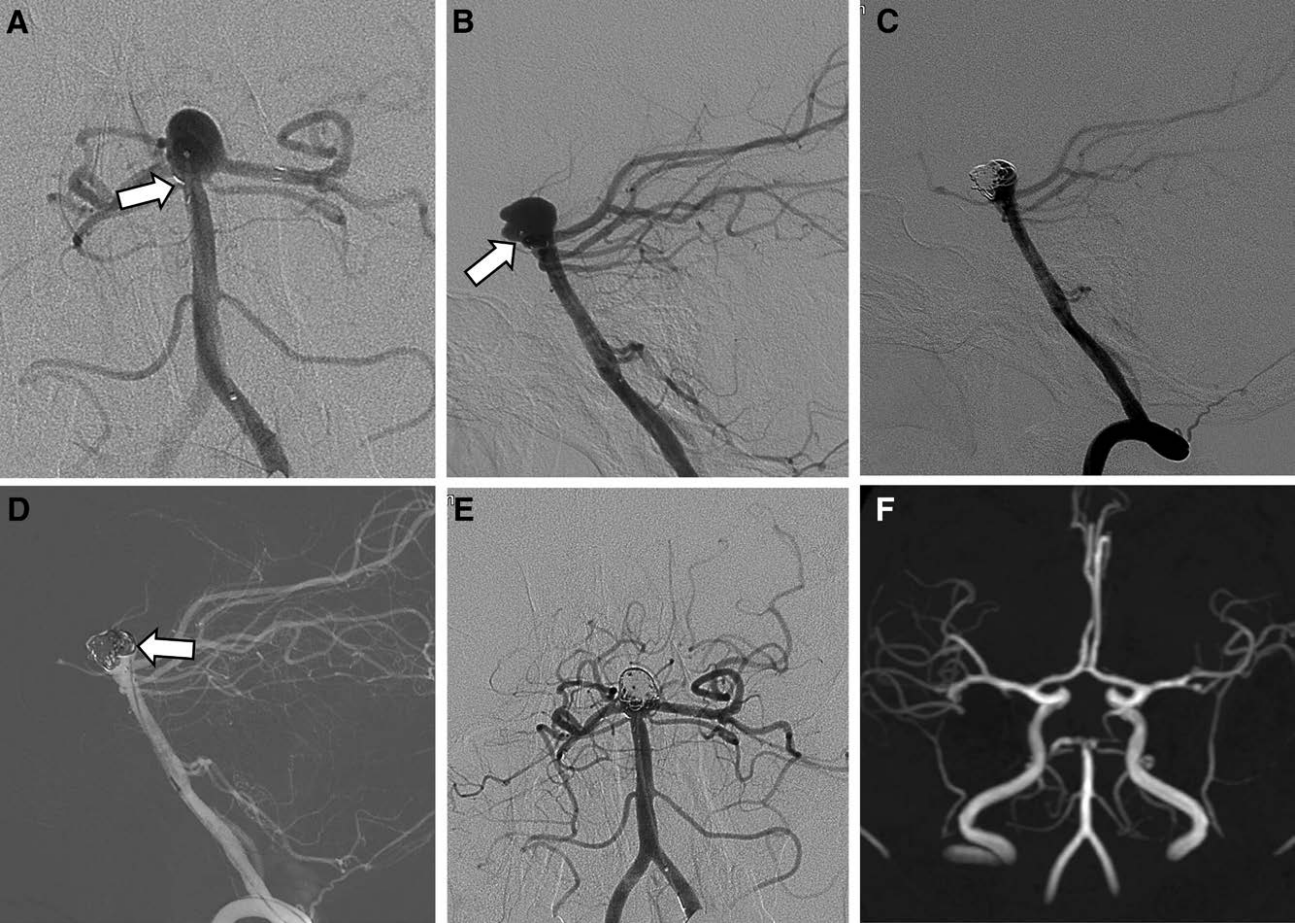
Shifting phenomenon of the jailed microcatheter anteriorly after horizontal stenting. (A) Left vertebral angiography after stent deployment showing proximal stent markers (arrow) fanned out near the orifice of the right superior cerebellar artery. (B) Lateral view showing the microcatheter tip (arrow) shifted towards the front of the deployed stent. (C) Insufficient coil filling in the posterior part of the aneurysm. (D) Additional coils inserted through the repositioned microcatheter with a posteriorly located tip (arrow). (E) Final angiography showing complete occlusion. (F) A 4-year follow-up time-of-flight MR angiography showing no recurrence of the treated aneurysm.

A 75-year-old woman was admitted for coil embolization of an incidentally detected basilar tip aneurysm. DSA was performed, revealing the dimensions of the aneurysm (width, 9.8 mm; neck, 7.5 mm; height, 7.7 mm). The left-sided P1 was more incorporated into the neck of this wide-neck aneurysm. Thus, we planned to perform stent-assisted coil embolization with the placement of an open-cell type stent into the left-sided P1. Under general anesthesia, a 6-Fr Chaperon guiding catheter was positioned in the left vertebral artery. A Headway microcatheter was placed in the aneurysm, and another Headway microcatheter was navigated to the P1 segment of the left PCA to deliver a Neuroform Atlas stent. DSA after the deployment of the 3 × 15 mm Neuroform Atlas stent (Stryker Neurovascular, Fremont, CA) showed horizontally fanned out stent markers near the orifices of the right PCA and right superior cerebellar artery (Fig. 2A). On the lateral view of DSA, the microcatheter tip within the aneurysm was shifted to the anterior side of the deployed stent (Fig. 2B). Coils were mostly inserted in the anterior part of the aneurysm (Fig. 2C). The microcatheter was then repositioned to the posterior remnant sac, and coiling was successfully completed (Fig. 2D). Final angiography revealed complete occlusion of the aneurysm (Fig. 2E). A total of 2000 U of heparin was administered during the procedure. Diffusion-weighted MR images taken 1 day after the procedure showed no ischemic changes. The patient was discharged without any change in neurological status. A follow-up MRI 4 years later showed no recurrence of the treated aneurysm (Fig. 2F).

## 3. Discussion

Stent-assisted coil embolization is widely used for the endovascular treatment of wide-neck aneurysms. Various protective stent techniques have been developed for endovascular coiling, including Y and X stents, horizontal stenting, herniation techniques using open-cell type stents, and bulging techniques with closed-cell type stents.^[1,2,4]^ Depending on the anatomical relationship between the aneurysm and the parent artery, different types of stents, including open-cell, closed-cell, and hybrid-cell designs, can be chosen.^[1,2]^ In the cases discussed here, we described the effectiveness of the Neuroform Atlas for antegrade horizontal stenting of basilar tip aneurysms and the positioning of a jailed microcatheter between the deployed stent via an antegrade approach.

Horizontal stenting can allow for denser coil packing and reduce the risk of thromboembolism compared to using multiple stents.^[4]^ Although horizontal stenting is designed to accommodate bifurcated site aneurysms with wide necks or acute angle efferent branches, issues regarding insufficient coil packing have still remained. In cases where there is microcatheter kickback after coiling with the trans-cell technique, reinserting the microcatheter through the stent struts can be challenging.^[4]^ To address this challenge, supplemental techniques such as using single or double jailed coil delivery microcatheters have been introduced. Kitahara et al reported that inserting double coil delivery microcatheters before retrograde horizontal Enterprise stenting was useful for achieving tight coil packing. Each jailed microcatheter tip was located at different positions in the aneurysm, allowing for sufficient coil packing.^[5]^

The antegrade horizontal stenting technique has been proposed as a simpler approach, using a single guiding system that can be applied regardless of anatomical conditions, allowing transcirculation.^[6]^ This technique involves combining antegrade stenting with the jailed microcatheter technique, where a length of the stent’s proximal part is left on the microcatheter equal to the coverage length of the aneurysm neck after catheterization. Once the partially stent-loaded microcatheter tip is positioned at the bifurcation site, the delivery wire and microcatheter are pushed to release the stent from the basilar artery. This manipulation can be advantageous when the stent length is short, but if it is too short, it may not sufficiently cover the aneurysm neck.

During antegrade horizontal stenting, a preselected microcatheter within a basilar top aneurysm may shift forwards or backwards. Consequently, the risk of compartmentalization increases as the jailed microcatheter is fixed to either side. This limitation necessitates repositioning of the microcatheter to the opposite side of the aneurysm for dense coil packing. From a different standpoint, when a catheter is shifted to one side after horizontal stenting, it ensures effective obliteration of the same side of the aneurysm, including the narrow space between the stent and the adjacent aneurysm wall. Ultimately, antegrade horizontal stenting can facilitate complete obliteration of the aneurysm through additional catheterization.

The most undesirable scenario of microcatheter shift during antegrade horizontal stenting is when the preselected microcatheter is pushed out and becomes lodged in the opposite P1 segment. To prevent this, it is advisable to position the microcatheter either anteriorly or posteriorly under the lateral view before stenting. Retrograde stenting, on the other hand, is not susceptible to this issue and is more suitable for intentional horizontal positioning. However, retrograde stenting can only be performed in accessible communicating arteries and requires a double guiding system.

From a technical standpoint, horizontal stenting using the antegrade approach may appear simpler than the retrograde approach. However, doubts may arise regarding the intentional positioning of the stent. A looping microcatheter technique has been introduced for antegrade horizontal stenting, involving the positioning of a steamed stent delivery microcatheter on the opposite side of the efferent vessel.^[7]^ The authors utilized the Neuroform Atlas, known for its high flexibility and compatibility with small profile microcatheters, to navigate through the looping microcatheter. However, navigating the microcatheter through looping and delivering the stent are technically challenging tasks, and there is a risk of vessel perforation for dissection during the procedure.

Conventional antegrade horizontal stenting may seem simple, but achieving the exact length of the stent can be challenging. The key to success lies in the precise landing point of the proximal stent.^[6]^ Antegrade horizontal stenting has been reported as an effective treatment not only for basilar top aneurysms but also for wide-neck aneurysms located at the large middle cerebral artery bifurcation and the large posterior communicating artery.^[2,6,7]^

The hybrid-cell design of the Neuroform Atlas stent facilitates antegrade horizontal stenting in several ways.^[2]^ First, unlike the Neuroform EX stent, which was first used for antegrade stenting for basilar tip aneurysm,^[6]^ the Neuroform Atlas stent has a closed-cell type at its proximal end. This design allows the proximal segment to move towards the opposite P1 side beyond the basilar artery when the wire is pushed. The closed cells also allow for easy re-crossing of the microcatheter to handle the landing point. Second, foreshortening and stent-kinking may be expected with pushing techniques. However, the Neuroform Atlas stent has a very short, single array of closed-cell columns, which may prevent these adverse events. Third, the junction between the open cell and closed cell acts as a joint, aiding in the stent’s ability to jump out. Finally, the reduced strut thickness compared to the Neuroform EX stent may enhance the safety of the procedure.

It is important to note that the length of the stent and exact landing points are critical, and the space at the bifurcation site needs to be large enough to accommodate the potential movement of the proximal stent. Antegrade horizontal stenting remains technically challenging, so this technique should only be used in select cases. In the 2 cases presented, successful coil placement in the aneurysm was achieved without any ischemic changes observed in the midbrain and thalamus on the MRI follow-up conducted on the next day.

This study has some limitations. First, the technical difficulty of antegrade stenting does not guarantee consistent success, particularly when using longer stents. Despite efforts to push the delivery stent wire and microcatheter, the proximal part of the stent may end up being deployed in the distal basilar artery. However, this issue can be referred to as the previously reported intentional herniation technique, which can also be considered one of the effective methods of stent-assisted coil embolization.^[8]^ This technique also involves pushing the delivery wire of an open-cell design stent and microcatheter. Second, predicting the position of the proximal markers after antegrade horizontal stenting is challenging. In the second case presented here, the proximal markers ended up around the superior cerebellar artery orifice. Finally, there may be insufficient coverage of the aneurysmal neck to prevent coil mass herniation or stent herniation of the proximal segment into the aneurysm. Thus, the length of the stent is critical in these cases.

## 4. Conclusion

Utilizing the Neuroform Atlas stent for antegrade horizontal stenting appears to be an effective and feasible approach for achieving complete obliteration of basilar tip aneurysms. The shifting phenomenon of a jailed microcatheter after antegrade horizontal stenting warrants attention, as this may present potential challenges that require further catheterization.

## Author contributions

**Conceptualization:** Byung Hyun Baek, Woong Yoon.

**Data curation:** Byung Hyun Baek, Tae Sun Kim, Byung Chan Lee.

**Formal analysis:** You Sub Kim, Yun Young Lee.

**Investigation:** You Sub Kim, Seul Kee Kim.

**Methodology:** Byung Hyun Baek, Woong Yoon, Tae Sun Kim, Byung Chan Lee, Chan Park.

**Project administration:** Tae Sun Kim.

**Validation:** Yun Young Lee.

**Writing – original draft:** Byung Hyun Baek.

**Writing – review & editing:** Woong Yoon, You Sub Kim, Seul Kee Kim, Yun Young Lee, Chan Park.
